# Investigation into the prevalence of enterotoxin genes and genetic background of *Staphylococcus aureus* isolates from retain foods in Hangzhou, China

**DOI:** 10.1186/s12866-023-03027-0

**Published:** 2023-10-17

**Authors:** Qi Chen, Gang Zhao, Wei Yang, Fuhong Chen, Yan Qi, Zhengqing Lou

**Affiliations:** 1 Hangzhou Traditional Chinese Medicine Hospital Affiliated to Zhejiang Chinese Medical University, 310000 Hangzhou, China; 2 Hangzhou Center for Disease Control and Prevention, 310021 Hangzhou, China

**Keywords:** *Staphylococcus aureus*, Staphylococcal enterotoxins, Gene cluster, Clonal complexes, *spa* types

## Abstract

**Background:**

*Staphylococcus aureus* expresses numerous toxins, many of which are strongly believed to be responsible for specific symptoms and even diseases, making it significant in the pathogenesis of human health. Enterotoxins, which are vital toxins, are associated with foodborne illnesses that manifest through symptoms like vomiting and diarrhea. In the present study, 264 *S. aureus* isolates obtained from various retail foods in Hangzhou, China were further investigated the profiles of enterotoxin genes and genetic backgrounds.

**Results:**

Approximately, 64.02% of the isolates from diverse sources contained at least one Staphylococcal Enterotoxin (SE) genes, displaying a total of 36 distinct combinations. Enterotoxin gene cluster (*egc*) encoded enterotoxin genes, normally designated by *seg*, *sei*, *sem*, *sen*, *seo* and *selu*, plus with *sep* were more frequently detected (33.73%, each). In contrast, *see*, *ses* and *set* were absent in any of the isolates tested. A total of 44 sequence types (STs), 20 clonal complexes (CCs) and 66 different staphylococcal protein A (*spa*) types (including six novel types) were identified among those 169 SE-positive isolates. Moreover, nineteen methicillin-resistant *Staphylococcus aureus* (MRSA) isolates were identified. The majority of those isolates belonged to the CC59-Scc*mec* IVa cluster and carried the *seb-sek-seq* gene cluster. The *egc* cluster, either coexisting with or without other enterotoxin genes, was observed in all isolates allocated into CC5, CC9, CC20, CC25, CC72 and ST672. Irrespective of the *spa* types and origins of the food, it appeared that *seh* was a distinct genetic element present in isolates belonging to the CC1 clonal lineage.

**Conclusions:**

The results not only proposed a suspected relationship between distribution of enterotoxigenic strains and genetic backgrounds, but also attributed the presence of novel enterotoxins to potential hazards in food safety.

**Supplementary Information:**

The online version contains supplementary material available at 10.1186/s12866-023-03027-0.

## Background

*Staphylococcus aureus*, an important bacterial pathogen in terms of human health, is responsible for a diverse array of infections, encompassing simple skin and soft tissue infections, as well as fetal septicaemia and osteomyelitis [1–3]. Staphylococcal food poisoning (SFP) is a common form of intoxication characterized by symptoms such as nausea, vomiting and abdominal pain [4]. It occurs when food items contaminated with *S. aureus* containing enough amounts of one or more enterotoxins [5, 6]. The illness is typically self-limiting in 1–3 days, but it can occasionally be serious, particularly in infants, the elderly and those with compromised immune systems [7]. *Staphylococcus aureus* is a prominent bacterial species known for its production of enterotoxins, causing numerous cases of foodborne illnesses globally [8–12]. It was estimated that more than 240,000 foodborne outbreaks per year was due to *S. aureus* in the United States alone [13]. From 2010 to 2020, *S. aureus* was found to be one of the leading pathogenic microorganisms in China, responsible for causing 577 outbreaks, 9092 cases of illness, 3715 hospitalizations and 2 death [14]. However, considering most cases of SFP experience self-recovery without hospitalization, the number of SFP cases may have been underestimated.

Staphylococcal enterotoxins (SEs), a superfamily of extracellular proteins with similar structures and functions, are often found to be responsible for SFP due to their robust tolerance to heat, low pH as well as their ability to withstand most proteolytic enzymes [6]. So far, more than 18 new types of SEs have been reported in addition to the five traditional enterotoxins (SEA ~ SEE) [6, 7]. Enterotoxins that failed to exhibit emetic activity or had not been evaluated in non-human primate models were classified as Staphylococcal Enterotoxin-like proteins (SEls), including SElJ, SElU, SElV, SElW, SElX and SElZ [6].

The majority of genes encoding SEs and SEls were located on mobile genetic elements (MGEs), while some of them appeared to coexist on plasmids, prophages, pathogenicity islands (SaPIs) and variable genetic islands. Genes encoding virulence factors and antibiotic resistance on MGEs can undergo horizontal transfer within *S. aureus*, thereby altering its pathogenicity and accelerating the genetic evolution of the strains in both animals and human hosts. Therefore, investigating the distribution of staphylococcal enterotoxin genes and MGEs would be advantageous in tracing the epidemiological origins and understanding the occurrence of virulence.

Various foods, particularly those containing starch and protein, have been reported to be vulnerable to be contaminated by *S. aureus* and subsequently SEs. Consequently, outbreaks of SFP have consistently been regional differences [6]. In this study, we evaluated the enterotoxin potential, especially the distribution of new SEs/SEls, together with the genetic diversity of *S. aureus* isolated from meat, milk, starch foods and fresh fruits/vegetables in Hangzhou, China, to probe into the possible relationship between the distribution of enterotoxin-coding genes and the genetic background of the strains.

## Results

### Prevalence and origins of *S. aureus*

Of the 2969 retail food samples, a total of 264 isolates, comprising 117 isolates (13.03%) from raw meat products, 85 isolates (9.22%) from cooked products, 50 isolates (7.04%) from starch foods, 10 isolates (6.33%) from drink, and 2 isolates (0.71%) from milk/milk products (seen in Table 1), were tested positive for *S. aureus* and further detected PCR of *nuc* gene. Moreover, among the 169 chosen isolates that exhibited SE positivity, a total of 19 isolates were identified as methicillin-resistant *Staphylococcus aureus* (MRSA) due to the presence of the *mecA* gene.

**Table 1:** Prevalence of *S. aureus* isolates in retail foods in Hangzhou

Source	No. of total samples	No. of isolates (%)	No. of SE-positive isolates (%)
Raw meat products	898	117 (13.03)	82 (70.09)
Cooked products	922	85 (9.22)	49 (57.65)
Starch food products	710	50 (7.04)	34 (68.00)
Drinks	158	10 (6.33)	2 (20.00)
Milk	281	2 (0.71)	2 (100.00)
Total	2969	264 (8.89)	169 (64.02)

### Enterotoxin gene profiles of *S. aureus*

**Fig. 1:**
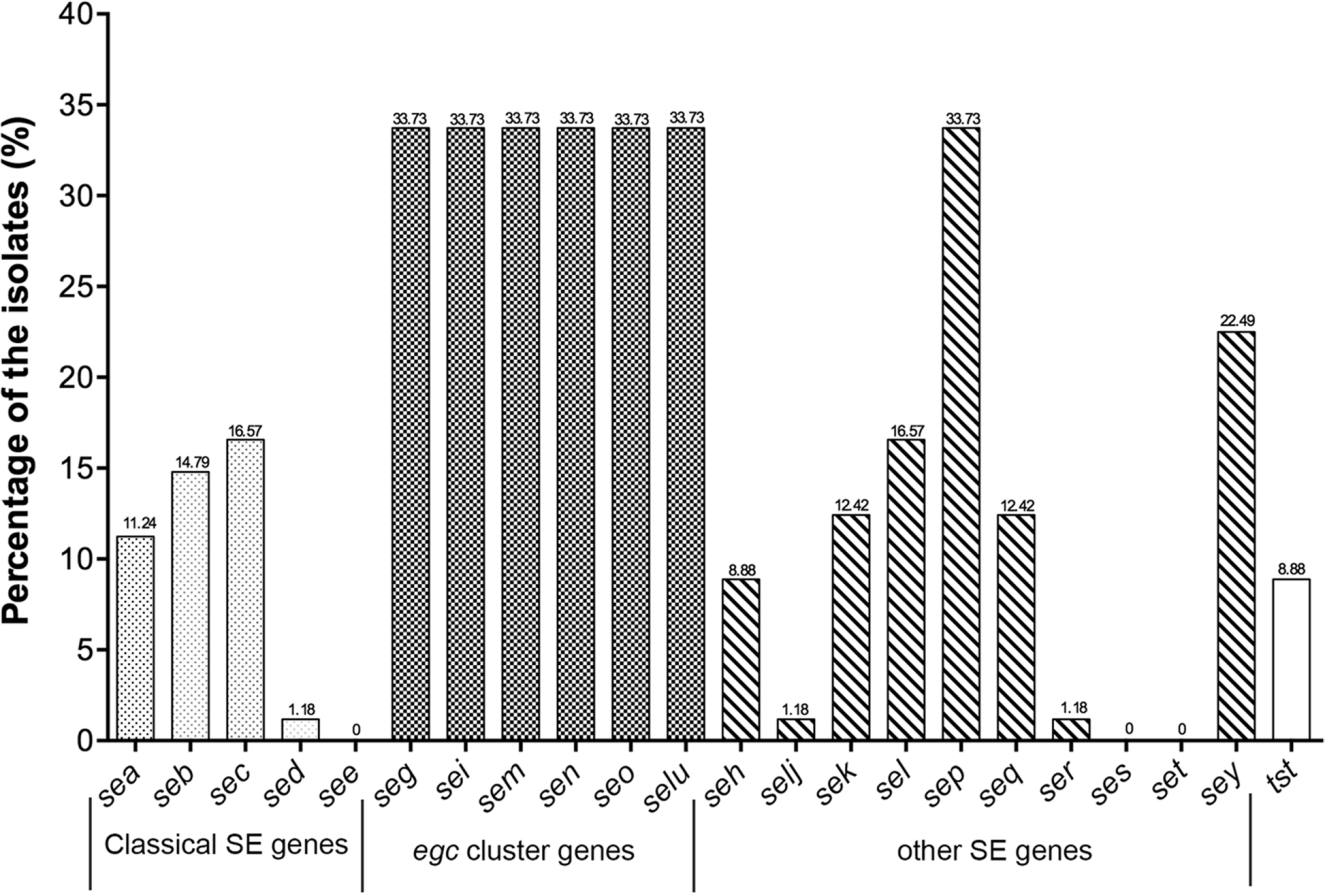
Distribution of the Staphylococcal toxin genes of *S. aureus* isolated from retail foods

Out of the 264 isolates analyzed, 169 of them (64.02%) harbored at least one enterotoxigenic gene. These isolates formed 36 distinct combinations (Table 2), with 4 of them carrying more than 10 SE genes. Only the isolates from vegetables and fruits had a lowest percentage of SE genes (6/18, 33.3%). The frequencies of every enterotoxin gene were presented in Fig. 1. The encoding genes for Enterotoxin E, S and T were not detected in all isolates. The most frequently detected genes were the Staphylococcal enterotoxin gene *sep* and *egc*-encoding genes, each found in 33.73% of the isolates. *sey* was detected in 22.49% of the isolates, followed by *sel* in 11.57%. The abundance of classical SE genes, including *sea* (11.24%), *seb* (14.79%), *sec* (16.57%) and *sed* (1.18%), which have frequently been linked to SFP outbreaks, was comparatively lower than that of many other newly discovered SEs or SEls. Notably, the most common combination of enterotoxin-encoding genes was *seg* + *sei* + *sem* + *sen* + *seo* + *selu*, accounting for 13.61% (23/169). There was no statistically significant difference among the catalog of food items in terms of the number of enterotoxin genes detected.

**Table 2:** Clonal Complexes, MLST, spa types and SE gene profiles of the enterotoxin-positive *S. aureus* isolates

CC	MLST	Spa tpye	Enterotoxin genes profile	SCC*mec* Type (No. of MRSA isolates)
CC7 (42)	ST7 (39)	t091 (32)	*sep* (28) *sep-sey* (2) *sec-sel-sep-tsst* (4)	
		t1943 (4)	*sep* (4)	
		t796 (1)	*sep* (1)	
		t867 (1)	*sep* (1)	
		**t20241** (1)	*sep* (1)	
	ST943 (1)	t091 (1)	*sep* (1)	
	ST4367 (1)	t236 (1)	*sec-sel-sep-tsst* (1)	
	ST7063(1)	t091 (1)	*sec-sel-sep-tsst* (1)	
CC1 (21)	ST1 (12)	t127 (6)	*seh* (2) *sec-sel-seh* (3) *sec-sel-seh-sek-seq* (1)	
		t114 (3)	*sec-sel-sek-seq-seh-tsst* (3)	SCC*mec* V (3)
		t1381 (1)	*seh* (1)	
		t1508 (1)	*sea-seb-sek-seq* (1)	
		t3324 (1)	*sea-sec-sel-seh-sek-seq-tsst* (1)	SCC*mec* V (1)
	ST2990 (3)	t091 (3)	*sec-sel-sey* (3)	
	ST1920 (2)	t7589 (1)	*seh* (1)	
		t2457 (1)	*seh* (1)	
	ST493 (1)	t091 (1)	*sep* (1)	
	ST573 (1)	t4938 (1)	*sec-sel-seg-sei-sem-sen-seo-selu* (1)	
	ST848 (1)	t409 (1)	*sec-sel-seg-sei-sem-sen-seo-selu -tsst* (1)	
	ST5881 (1)	t1407 (1)	*seh* (1)	
CC5 (17)	ST5 (8)	t548 (4)	*sec-sel-seg-sei-sem-sen-seo-selu* (3) *sed-selj-ser-sec-sel-seg-sei-sem-sen-seo-selu-seh* (1)	
		t002 (2)	*sec-sel-sed-selj-ser-sec-sel-seg-sei-sem-sen-seo-selu-sey* (2)	
		t311 (1)	*sec-sel-seg-sei-sem-sen-seo-selu* (1)	SCC*mec* V (1)
		**t20441** (1)	*sec-sel-seg-sei-sem-sen-seo-selu* (1)	
	ST965 (5)	t062 (5)	*sec-sel-seg-sei-sem-sen-seo-selu* (3) *sea-sec-sel-seg-sei-sem-sen-seo-selu* (2)	
	ST950 (1)	t895 (1)	*sec-sel-seg-sei-sem-sen-seo-selu* (1)	
	ST6427 (1)	t954 (1)	*sec-sel-seg-sei-sem-sen-seo-selu* (1)	
	**ST7563** (1)	t954 (1)	*sed-selj-ser-sec-sel-seg-sei-sem-sen-seo-selu* (1)	
	ST2144 (1)	t1107 (1)	*sec-sel-seg-sei-sem-sen-seo-selu* (1)	
CC59 (14)	ST59 (14)	t437 (10)	*seb-sek-seq-sey* (9) *sea-seb-sek-seq-sey* (1)	SCC*mec* IVa (7)
		t3523 (1)	*seb-sek-seq-sey* (1)	SCC*mec* IVa (1)
		t3527 (1)	*seb-sek-seq-sey* (1)	SCC*mec* IVa (1)
		t3590 (1)	*seb-sek-seq-sey* (1)	SCC*mec* IVa (1)
		t16156 (1)	*sea-seb-sek-seq* (1)	SCC*mec* IVa (1)
CC20 (13)	ST2631 (7)	t164 (7)	*sec-sel-seg-sei-sem-sen-seo-selu-sey* (7)	
	ST1281 (4)	t164 (3)	*sec-sel-seg-sei-sem-sen-seo-selu-sey* (3)	
		t996 (1)	*sec-sel-seg-sei-sem-sen-seo-selu-sey* (1)	
	ST1921 (1)	t164 (1)	*sec-sel-sec-sel-seg-sei-sem-sen-seo-selu-sey-tsst* (1)	
	ST20 (1)	t3277 (1)	*sec-sel-seg-sei-sem-sen-seo-selu-sey* (1)	
CC6 (13)	ST6 (12)	t701 (11)	*sea* (10) *sea-seb* (1)	
		**t20424** (1)	*sea* (1)	
	ST1551 (1)	t304 (1)	*sea* (1)	
CC25 (9)	ST25 (8)	t078 (5)	*sec-sel-sec-sel-seg-sei-sem-sen-seo-selu* (4) *sec-sel-seg-sei-sem-sen-seo-selu* (1)	
		t353 (1)	*sec-sel-seg-sei-sem-sen-seo-selu-seh* (1)	
		t1102 (1)	*seb-sec-sel-seg-sei-sem-sen-seo-selu* (1)	
		**t20426** (1)	*seb- sec-sel-seg-sei-sem-sen-seo-selu* (1)	
	ST2797 (1)	t081 (1)	*seb- sec-sel-seg-sei-sem-sen-seo-selu* (1)	
CC72 (8)	ST72 (7)	t148 (4)	*sec-sel-seg-sei-sem-sen-seo-selu* (3) *sec-sel- sec-sel-seg-sei-sem-sen-seo-selu-tsst* (1)	
		t901 (1)	*sec-sel-seg-sei-sem-sen-seo-selu* (1)	
		t3092 (1)	*sec-sel-seg-sei-sem-sen-seo-selu* (1)	
		t3169 (1)	*sec-sel-sec-sel-seg-sei-sem-sen-seo-selu-tsst* (1)	
	ST544 (1)	t126 (1)	*sec-sel-seg-sei-sem-sen-seo-selu* (1)	
CC88 (7)	ST88 (7)	t2592 (4)	*sep* (4)	
		t3622 (1)	*sep* (1)	SCC*mec* II (1)
		t10777 (1)	*sep* (1)	
		**t20425** (1)	*sep* (1)	
CC188 (5)	ST188 (4)	t189 (3)	*seb* (3)	
		t2883 (1)	*seb* (1)	
	**ST7562** (1)	t189 (1)	*seb* (1)	
CC8 (3)	ST630 (2)	t377 (1)	*sec-sel* (1)	
		t4047 (1)	*sey* (1)	
	ST2416 (1)	t024 (1)	*sek-seq-sep* (1)	
CC9 (3)	ST9 (2)	t899 (2)	*sec-sel-seg-sei-sem-sen-seo-selu-sey* (1) *sec-sel-seg-sei-sem-sen-seo-selu -sep-sey* (1)	SCC*mec* IVb (2)
	ST2423 (1)	t15045 (1)	*sec-sel-seg-sei-sem-sen-seo-selu-sey* (1)	
CC1148 (3)	ST1148 (3)	t16825 (3)	*sep* (3)	
CC22 (2)	ST22 (2)	t309 (2)	*sec-sel-seg-sei-sem-sen-seo-selu* (2)	
CC12 (1)	ST12 (1)	t213 (1)	*sep* (1)	
CC15 (1)	ST15 (1)	t2413 (1)	*sec-sel-tsst* (1)	
CC101 (1)	ST101 (1)	t2078 (1)	*sep* (1)	
CC121 (1)	ST946 (1)	**t20422** (1)	*seb- sec-sel-seg-sei-sem-sen-seo-selu-sey* (1)	
CC398 (1)	ST398 (1)	t1928 (1)	*sec-sel* (1)	
CC522 (1)	ST522 (1)	t5428 (1)	*sec-sel-y-tsst* (1)	
Singletons (3)	ST672 (3)	t4336 (3)	*sec-sel-seg-sei-sem-sen-seo-selu* (3)	

### Molecular typing of SE-positive *S. aureus*

Using the Multi-locus Sequence Typing Method, a total of 169 SE-positive *S. aureus* isolates were identified. These isolates were classified into 44 sequence types (STs), which were further grouped into 20 clonal complexes (CCs) and 3 singletons (refer to Table 2) based on eBURST analysis. Two isolates 18–57 and 20–38 were assigned new STs (ST7562 and 7563) after registrations to the MLST database. The most frequently detected STs was ST7 (39/169, 23.08%), followed by ST59 (14/169, 8.28%). The study followed ST1 and ST6, with each accounting for 7.10% (12 out of 169). ST5 and ST25 were detected at a rate of 4.73% each (seen in Table 2). 66 different *spa* types including six novel *spa* types (t20421, t20422, t20424, t20425, t20426, t20441) that were not listed in the Ridom StaphType database, were found among those 169 *S. aureus* isolates. t091 was the most prevalent *spa* type, accounting for 38 out of 169 isolates (22.49%). t164 and t701 accounted for 11 isolates each out of 169 (6.51% each), respectively. t437 accounted for 10 out of 169 (5.92%). Combining the STs and *spa* types, ST7 - t091 was the predominant molecular types (32/169, 18.93%) of all isolates, whereas ST59 - t437 was the most frequently detected among MRSA isolates. The majority of isolates showed low consistency between MLST and *spa* types in this study. For example, 39 isolates belonged to the same ST7 but were divided into five different *spa* types (t091, t1943, t796, t867, t20241). Meanwhile, some strains had the same *spa* type but diverse MLST types (t164, t954, t189) and were associated with different Clonal Complex (CC20-t164, CC5-t954, CC188-t189).

**Fig. 2:**
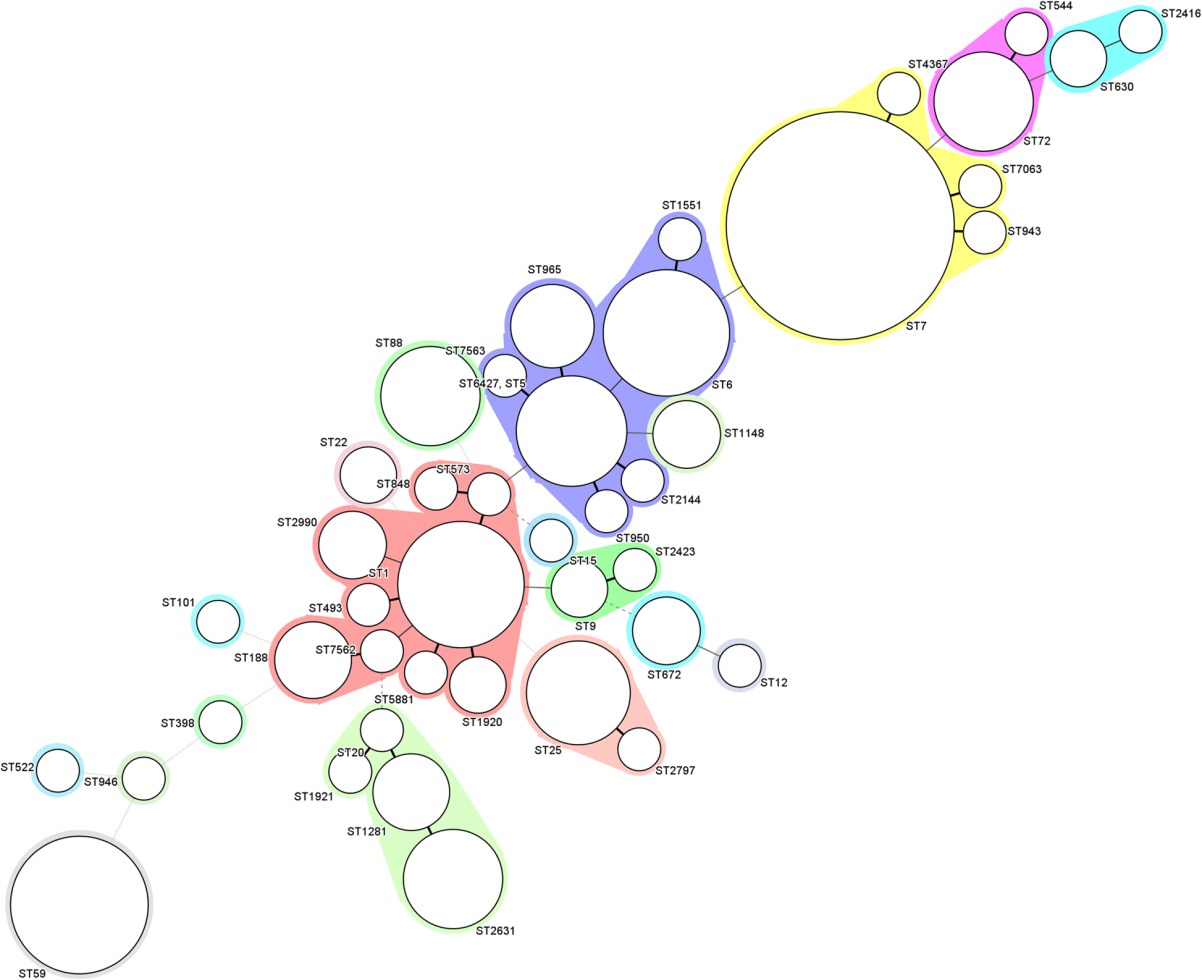
Minimum spanning tree of MLST types for 169 enterotoxin-positive *S. aureus* isolates. Each circle represents one ST, the size of which accounts for the numbers of strains with the same ST, while the gray zones around some STs means that these genotypes belong to the same clonal complex

The relatedness among the strains was further examined by constructing a phylogenetic tree based on the seven-allelic combinations of MLST (Fig. 2.). All STs were divided into three distinct cladograms (named as A, B and C). Cladogram A was a complex composition, including CC1 (ST1, ST2990, ST1920, ST493, ST573, ST848, ST5881), CC188 (ST188, ST7562), CC9 (ST9, ST2423), CC25 (ST25, ST2797), CC59 (ST59), CC20 (ST20, ST1921, ST1281, ST2631) and some relevant single-locus variant, and the most relevant Clonal Complex 1 occupied the vast majority. Cladogram B contained CC5 (ST5, ST965, ST950, ST2144, ST7563, ST6427), CC6 (ST6, ST1551) and CC88 (ST88). Cladogram C consisted of CC7 (ST7, ST943, ST7063, ST4367), CC72 (ST72, ST544) and CC8 (ST630, ST2416).

## Discussion

Quite a few studies had investigated the proportion of enterotoxigenic genes in various kinds of food and clinical samples, particularly in light of increased awareness of the risks associated with emerging novel types of SEs and SEls. However, the genetic background and information of SE-positive *S. aureus* strains circulating through food chain in Hangzhou City is limited. In this study, 64.2% (169/264) isolates from different retail foods harbored one or more *se*/*sel* genes, averaging three genes per isolate. The distribution of individual enterotoxin genes varied among previous articles mainly due to their diverse geographical locations. However, approximately 50–80% of isolates carried at least one *se*/*sel* gene [15–17], which was consistent with our results. The most frequently occurred genes were *sep*, *seg*, *sei*, *sem*, *sen*, *seo* and *selu* (33.79%, each) in our study. Higher The prevalence of *egc* in food- and clinic-derived *S. aureus* isolates has been increasingly common in recent years, with prevalence rates ranging from 50% to a staggering 97.8% [18–21]. The significance of *egc* enterotoxins in terms of food safety has been highlighted, as there have been reports of *S. aureus* harboring *egc* enterotoxin genes without producing classical SEs in Japan, USA, Switzerland, Romania [6, 22, 23]. Recently, we described a foodborne outbreak due to *S. aureus* (new sequence type 7591) harboring *egc*-related genes in the absence of any classical SEs in China firstly [Int J Infect Dis. 2023 Oct;135:132-135]. Moreover, a total of five *S. aureus* strains (18–98, 19–39, 20–33, 20–34, 20–106) in this study showed identical *SmaI*-digested PFGE dendrograms with those in SFP and all of them were assigned to CC72 with different but pretty close *spa* types. A phylogenetic tree of these CC72 isolates based on the results of Whole-genome sequencing indicated a close genetic background between them. The high prevalence of *egc* in clinical isolates supported the hypothesis that *egc* may provide a selective advantage during infection [24] and play an important role in long-term persistence in the infant gut [25]. As foods commonly serve as a reservoir for clinical *S. aureus*, investigation of their prevalence and emergence of enterotoxin genes was of great importance.

The enterotoxin gene cluster (*egc*), an operon encoding a variety of *se*/*sel* genes, was initially discovered to comprise *seg*, *sei*, *sem*, *sen*, *seo* and two pseudogenes (*Ψent1* and *Ψent2*) [24, 26]. Subsequent to that, the composition and organization of the *egc* loci can vary due to random duplication, removal, or further variation, resulting in six general groups (*egc*1 to 6) [27]. All isolates carrying the *egc* cluster consisted of the universally present six genes, with or without any other enterotoxin genes. *selu* was suspected to be undectable due to its uncertain emetic activity in some studies. What’s more, the *egc* cluster was observed in all CC5, CC9, CC20, CC25, CC72 and ST672, but occasionally present in CC1, all of which (except for ST672) were reported in other previous studies but with various spa types [28–30]. These data suggested that a close connection between the *egc* cluster and the clonal genetic background. Differentiating the polymorphism of *egc* loci and further analysis of genetic location will be the next direction of our work.

Mobile genetic Elements (MGEs) are a collection of sizable, mobile DNA segments that serve as a reservoir for antibiotic genes. Moreover, they also function as the breeding ground for enterotoxin genes, leading to a non-random distribution of these genes in various individual studies.

*S. aureus* pathogenicity islands (SaPIs) are found to be widespread in *S. aureus*, and potentially in other Staphylococcus species. These islands contain various virulence genes such as SE- and SE-like genes, biofilm-associated genes, and genes responsible for drug resistance and host range [31, 32]. To date, six types of superantigen genes, which include *seb*, *sec*, *sek*, *sel*, *seq* and *tst* (firstly designed as *sef*) were found to present on SaPIs and always exist in varying combinations [33–36]. Examples of SaPI bov1, SaPI m1/n1, SaPI 6811 and SaPI J50 show the presence of *tst* either with *seq* and *sek* or without any toxin genes. In both SaPI 5 and SaPI j11, *sek* and *seq* were simultaneously detected. Additionally, in SaPI 3, *sek* and *seq* were found to coexisted with *seb*. Intriguingly, the presence of *seb* was also detected in uncommon SaPIs, like SaPI vm10, SaPI ishikawa11, SaPI ivm60, SaPI no10, SaPI hirosaki4, SaPI NN54 and SaPI PM1 [35, 37]. Based on the presence of enterotoxin genes on SaPIs, *sec* and *sel*, as well as *seq* and *sek*, could be separate combinations, which aligns with the findings of our study. Sixteen isolates carrying *sec* and *sel* without *tst* gene could potentially contain SaPI mw2. Two isolates harboring *sek* and *seq* without *sea*, *seb* or *tst* gene were suspected to have either SaPI 5 or SaPI j11. Identifying the specific SaPIs of the latest isolates with the *sec-sel* and *sek-seq* genotype solely based on the PCR results of SE profiles proved to be challenging. Furthermore, previous studies have shown that the *S. aureus* genome may contain one or more SaPIs [38, 39], which contributed to increased uncertainty. Whole genome sequencing, comprehensive nucleotide fragment analyses, or other low-cost and time-saving method (for example, LA-PCR based SaPI scanning) [35] are needed to comprehensively analyze the diversity of SaPIs in our future work.

The putative transposase gene, *seh*, has been reported to be located at immediately downstream of the type IV Staphylococcal Cassette Chromosome Element (SCC*mec*) and is responsible for stabilizing the integration of SCC*mec* IV [40, 41]. In this study, 3 of 15 isolates that tested positive for *seh* were assigned to ST1-t114-SCC*mec* IVg and co-expressed *sec-sel-tst-sek-seq* genes. Notably, this strain was implicated in causing SFP in China during 2014–2016, underscoring the significance of monitoring the presence of *S. aureus* in the food supply chain [42]. Among them, the genotype ST1-t127 (6/15) was also prevalent in China and other countries [29, 43, 44], indicating that it would be the main genotype of CC1 [44]. Remarkably, the presence of the *seh* gene was found to be associated with CC1 irrespective of the *spa* types and food origins (Table 2). This observation was consistent with prior research findings [44–46].

MRSA has emerged as a formidable and life-threatening pathogen since its first appearance in 1961, primarily due to its variable resistance to multiple drugs [47]. The prevalence of MRSA in food, particularly in meat and meat products, has shown an upward trend over the past decade [48–51].This study identified 19 MRSA isolates through detecting *mecA* gene, which belonged to five kinds of clonal complexes, namely CC1, CC5, CC9, CC59 and CC88. These clonal complexes have been previously reported and have identical profiles of enterotoxin genes [44, 46]. ST59-t437 MRSA isolates were the most frequent type identified in our study, supported by the consistent genetic information obtained from previous isolates found in food and hospitalized patients in China [44, 52, 53]. It was noted that MRSA isolates belonged to ST59 but allocated to other *spa* types were identified. Except for t16156, t3523, t3527 and t3590 had only one spa repeat difference from t437. Thus, it can be assumed that these strains emerged from the ancestor t437-ST59 through tandem deficiency and insertion. All ST59 isolates carried SCC*mec* IVa, *sek*, and *seq*, with occasional presence of *sea* and *pvl*. Previous studies in molecular epidemiology have demonstrated that CC59- SCC*mec* IV- t437 clone was the prevailing lineage of CA-MRSA in China and Vietnam [54]. The relatively high prevalence of CC59-t437 and its closely related MRSA lineage in our study suggested that food-origin SCC*mec* IVa-MRSA was probably to be the primary source of CA-MRSA in China, specifically in the city of Hangzhou.

This study was the first systematic surveillance of *S. aureus* isolated from foods in the city of Hangzhou, in which the genetic background of the SE-positive strains were determined. However, the study was conducted in local regions, which may not provide a complete representation of the potential enterotoxigenic *S. aureus* from food chain. Therefore, further research should be conducted to comprehensively evaluate the prevalence and genetic features of SE-positive *S. aureus*, including other cities and other food sources.

## Conclusion

In summary, our present study illustrated the distribution of enterotoxin genes and the genetic background diversity of *S. aureus* isolated from various food products being sold in Hangzhou, China. A total of 64.02% of *S. aureus* isolates were found to carry one or more SE/SEl genes and they were allocated into 44 sequence types and 66 different *spa* types. Our results verified the simultaneous occurrence of *sec-sel*, *sek-seq*, *sed-sej* and *seg-sei-sem-sen-seo-selu* regardless of the tandem variants. Notably, some special SE-positive *S. aureus* assigned to same CCs had a certain profile of enterotoxin genes, indicating the utility and discriminatory power of MLST as a molecular tool for estimating the presence of potential SE genes and their related clusters.

## Materials and methods

### Sample collection

A total of 2969 food samples, purchased from local markets in 15 districts of Hangzhou over a three-year period (January 2018 to December 2020), were randomly collected for *S. aureus* isolation. Collected samples were tightly sealed with sterile plastic wrap, stored in a cold box below 4 ℃, transported to accredited laboratory, and analyzed microbiologically within 24 hours. The tested food samples were divided into 5 categories, i.e., raw meat products (fresh beef, pork, mutton, duck and chicken samples), cooked products (processed meat products, ready-to-eat vegetables, soybean products, egg products and seafood), starch food products (sandwiches and traditional Chinese pastries), milk (cartons of milk sold on the supermarkets) and drinks (fresh juice and tea).

### Isolation and identification of *S. aureus*

The qualitative test for *S. aureus* in food samples was performed in accordance with GB 4789.10–2016, which is the National Food Safety Standard of China for the examination of *S. aureus* in food [55]. Briefly, approximately 25 g/ml of food items, added with 225 ml Tryptic Soy Broth supplemented with 7.5% NaCl (Huankai, Guangdong, China), were homogenized before incubation at 37 ℃ for 18–24 h. A loopful of the enriched cultured medium were transferred to blood agar plates and Baird-Parker plates for another 24–48 h. Putative *S. aureus* clone with a black color on BP plates or a clear hemolysis ring in blood agar plates was tested for coagulase activity test and further confirmed by the Vitek 2 compact system (bioMerieux, Marcy-1’Etoile, France). Furthermore, all the isolates were identified to the species level by PCR for the *nuc* gene.

## Detection of Staphylococcal enterotoxin genes

Total bacterial DNA was extracted from 1 ml cultured MH broth (Huankai, Guangdong, China) of isolates using the DNase Blood and Tissue Kit (Qiagen, Dusseldorf, Germany) under the guidelines of the manufacturer. PCR method was conducted for all isolates to determine the presence of genes encoding 22 staphylococcal enterotoxins plus TSST. PCR procedures and the visualization of amplicons were performed as described in our earlier published research [56]. The positive control for *nuc* genes was *Staphylococcus aureus* ATCC25923, while *S. epidermidis* ATCC12228 served as negative controls. Positive controls for toxin genes included the following strains: *Staphylococcus aureus* ATCC8095 (*sea, sed, selj, sek, seq, and ser* gene), *Staphylococcus aureus* ATCC14458 (*seb* gene), *Staphylococcus aureus* ATCC19015 (*sec* gene), *Staphylococcus aureus* ATCC27644 (*see* gene), *Staphylococcus aureus* 19–39 (*sec, sel, tsst, seg, sei, sem, sen*, *seo* and *selu* genes), *Staphylococcus aureus* 19–52 (*seh*, *seq, sek* gene), and *Staphylococcus aureus* 18–66 (*sep* gene), *Staphylococcus aureus* 18 − 11 (*sey* gene). *Staphylococcus aureus* strains 18 − 11, 18–66, 19–39, and 19–52 were isolated in this study and subjected to whole genome sequencing.

### Multi locus sequence typing and *spa* typing

Primer spa-1113f (5’-TAA AGA CGA TCC TTC GGT GAG C-3’) and spa-1514r (5’-CAG CAG TAG TGC CGT TTG CTT-3’) were used for the polymorphic X region of *spa* gene. MLST scanning was based on the sequences of the following seven housekeeping genes: *arcC*, *aroE*, *glpF*, *gmk*, *pta*, *tpi* and *yqiL*. The PCR amplification conditions were described at previous study [15]. Sequence type (ST) was determined by multilocus sequence typing (MLST) scheme and assigned to clonal complex (CC), according to the PubMLST (https:/pubmlst.org/organisms/ staphylococcus aureus). The clonal complex (CC) analysis was determined using the eBURST algorithm [57]. The *spa* types were assigned on the Spa Server Website (http://spaserver2.ridom.de/). The minimum spanning tree (MST) was constructed with Bionumerics 8.0 software (Applied Maths, Sint-Martens-Latem, Belgium).

### Supplementary Information

**Additional file 1: Table 1.** Primers used for detection of Staphylococcal enterotoxin genes

## Data Availability

Not applicable.
