# Supplementary material for: Investigation into the prevalence of enterotoxin genes and genetic background of Staphylococcus aureus isolates from retain foods in Hangzhou, China
Source: BMC Microbiol. 2023 Oct 17;23:294. doi: 10.1186/s12866-023-03027-0 (PMC10580612; doi:10.1186/s12866-023-03027-0)
Supplement: Supplementary file 1 — Additional file 1: Table 1. Primers used for detection of Staphylococcal enterotoxin genes [file 12866_2023_3027_MOESM1_ESM.docx]

Table 1 Primers used for detection of Staphylococcal enterotoxin genes.

| Gene | Nucleotide sequence (5’→3′) | Amplicon size (bp) | Reference |
| --- | --- | --- | --- |
| *sea* | CCTTTGGAAACGGTTAAAACG | 127 | [1] |
|  | TCTGAACCTTCCCATCAAAAAC |  |  |
| *seb* | TCGCATCAAACTGACAAACG | 477 | [1] |
|  | GCAGGTACTCTATAAGTGCCTGC | 271 |  |
| *sec* | CTCAAGAACTAGACATAAAAGCTAGG |  | [1] |
|  | TCAAAATCGGATTAACATTATCC |  |  |
| *sed* | CTAGTTTGGTAATATCTCCTTTAAACG | 319 | [1] |
|  | TTAATGCTATATCTTATAGGGTAAACATC |  |  |
| *see* | CAGTACCTATAGATAAAGTTAAAACAAGC | 178 | [1] |
|  | TAACTTACCGTGGACCCTTC |  |  |
| *seg* | CTGAATAAGTTAGAGGAGGTTT | 592 | [2] |
|  | TCTTTAGTGAGCCAGTGTCTTG |  |  |
| *sei* | TATGTATGGAGGGGTCACTTTATCA | 345 | [2] |
|  | AAGGAACTTACAGGCAGTCCATCTC |  |  |
| *sem* | TTACTTTAGCGGGTGATT | 318 | [2] |
|  | CTTGCCCTGTTCCTGTAT |  |  |
| *sen* | TAAACGGAGGAGTTACGATA | 301 | [2] |
|  | AACTCTGCTCCTACTGAACC |  |  |
| *seo* | TCTTTAGAAATCGCTGATGA | 214 | [3] |
|  | TGTGTAAGAAGTCAAGTGTAG |  |  |
| *selu* | AAACATTAAAGCCCAAGAG | 243 | [2] |
|  | ACACCGCCATACATACAC |  |  |
| *seh* | ACTATCATTTCCATTCTAACTAC | 431 | [2] |
|  | AATCATTGCCACTATCACCTTG |  |  |
| *selj* | TAACCCCTTTAGTTTACAGCGATAG | 476 | [2] |
|  | AATGGTTACTTTTTTCTTATTTGTT |  |  |
| *sek* | ATGGATCAATGGAAATCACAAA | 271 | [2] |
|  | TGGTAACCCATCATCTCCTGTG |  |  |
| *sel* | TAACGGCGATGTAGGTCCAGG | 383 | [3] |
|  | CATCTATTTCTTGTGCGGTAAC |  |  |
| *sep* | TGATTTATTAGTAGACCTTGG | 396 | [3] |
|  | ATAACCAACCGAATCACCAG |  |  |
| *seq* | AATCTCTGGGTCAATGGTAAGC | 122 | [1] |
|  | TTGTATTCGTTTTGTAGGTATTTTCG |  |  |
| *ser* | GGATAAAGCGGTAATAGCAG | 166 | [3] |
|  | GTATTCCAAACACATCTAAC |  |  |
| *ses* | TTCAGAAATAGCCAATCATTTCAA | 195 | [4] |
|  | CCTTTTTGTTGAGAGCCGTC |  |  |
| *set* | GGTGATTATGTAGATGCTTGGG | 170 | [4] |
|  | TCGGGTGTTACTTCTGTTTGC |  |  |
| *sey* | CAATGTACGGACAGTGCTCTACAA | 189 | [5] |
|  | TGACCGTTAACAAACAAGTTCATTC |  |  |
| *tst* | AAGCCCTTTGTTGCTTGCG | 445 | [1] |
|  | ATCGAACTTTGGCCCATACTTT |  |  |

1. Becker K, Roth R, Peters G: Rapid and specific detection of toxigenic Staphylococcus aureus: use of two multiplex PCR enzyme immunoassays for amplification and hybridization of staphylococcal enterotoxin genes, exfoliative toxin genes, and toxic shock syndrome toxin 1 gene. J Clin Microbiol 1998, 36(9):2548-53.

2. Tang J, Tang C, Chen J, Du Y, Yang XN, Wang C *et al*: Phenotypic characterization and prevalence of enterotoxin genes in Staphylococcus aureus isolates from outbreaks of illness in Chengdu City. Foodborne Pathog Dis 2011, 8(12):1317-20.

3. Omoe K, Hu DL, Takahashi-Omoe H, Nakane A, Shinagawa K: Comprehensive analysis of classical and newly described staphylococcal superantigenic toxin genes in Staphylococcus aureus isolates. FEMS Microbiol Lett 2005, 246(2):191-8.

4. Ono HK, Omoe K, Imanishi K, Iwakabe Y, Hu DL, Kato H *et al*: Identification and characterization of two novel staphylococcal enterotoxins, types S and T. Infect Immun 2008, 76(11):4999-5005.

5. Ono HK, Sato'o Y, Narita K, Naito I, Hirose S, Hisatsune J *et al*: Identification and Characterization of a Novel Staphylococcal Emetic Toxin. Appl Environ Microbiol 2015, 81(20):7034-40.
